# Transcatheter Mitral Valve-in-Valve Implantation Applying a Long Pre-Curved Sheath for Patients with Degenerated Bioprosthetic Mitral Valve

**DOI:** 10.31083/j.rcm2402050

**Published:** 2023-02-06

**Authors:** Yang Liu, Mengen Zhai, Chennian Xu, Lanlan Li, Yu Mao, Yanyan Ma, Ping Jin, Wuchao Xue, Jian Yang

**Affiliations:** ^1^Department of Cardiovascular Surgery, Xijing Hospital, Air Force Medical University, 710032 Xi’an, Shaanxi, China; ^2^Department of Cardiovascular Surgery, General Hospital of Northern Theater Command, 110016 Shenyang, Liaoning, China

**Keywords:** mitral valve, transcatheter valve implantation, degenerated bioprosthesis, valve-in-valve procedures, intervention

## Abstract

**Backgrounds:**

Percutaneous transseptal transcatheter mitral valve-in-valve implantation (TMViV) has become an alternative minimally invasive treatment choice for patients with degenerated mitral bioprosthesis and high surgical risk. However, transseptal approach is more technically challenging than transapical approach in TMViV procedures.

**Objective:**

The objective of this study was to introduce the experience of applying long pre-curved sheaths in transseptal TMViV procedures and to evaluate the effect of long pre-curved sheath techniques in TMViV procedures.

**Methods:**

Between January 2020 and December 2021, 27 patients with degenerated bioprosthetic mitral valve underwent TMViV procedures using a balloon-expandable valve via the transseptal approach. The regular 14/16F expandable sheath were used for low-profile delivery in first 10 cases, and 22F long pre-curved sheath were used in the next 17 cases during procedures. We retrospectively reviewed the catheter techniques, perioperative characteristics, and prognosis. The median follow-up time was 12 (1–21) months. To further scrutinize our data, we divided the group into the early 10 patients using 14/16F expandable sheath and the subsequent 17 patients with long pre-curved sheath in order to assess the impact of different sheaths and procedural details on outcomes.

**Results:**

Procedural success was obtained in all patients with no in-hospital mortality. Seventeen patients received 26 mm prostheses; the remaining ten patients received 29 mm prostheses. Post balloon dilatation was performed in one case. Total procedure time was (96.1 ± 28.2) min, the fluoroscopic time was (27.4 ± 6.5) min, and total contrast volume was (50.7 ± 10.1) mL. One patient received blood transfusion because of hemorrhage at the femoral puncture site. One patient received a permanent pacemaker implantation due to high-degree atrioventricular block at postoperative day 3. There were no other major post-procedure complications and the median length of hospital stay was 4 days. Twenty-five (92.6%) patients improved by ≥1 New York Heart Association (NYHA) functional class at 30 days. In subsequent sub analysis, there were shorter procedural time [(85.2 ± 24.3) vs. (115.2 ± 25.6) min, *p* = 0.0048] and shorter fluoroscopic time [(24.3 ± 5.2) vs. (31.3 ± 5.1) min, *p* = 0.0073] in cases with the long pre-curved sheath than ones with regular expandable sheath. The iatrogenic atrial septal defect (ASD) closure was performed because of the transeptal large right to left shunt in 2 cases with regular expandable sheath, but no patient needed intraoperative ASD closure in cases with the long pre-curved sheath.

**Conclusions:**

Transseptal TMViV using long pre-curved sheath could simplify transseptal approach with reliable outcomes for patients of degenerated mitral bioprosthesis.

## 1. Introduction

Transcatheter mitral valve implantation (TMVI) represent a new treatment option in patients with degenerated bioprostheses, failed annuloplasty rings, and severe mitral annular calcification at high risk for conventional mitral valve surgery [1, 2]. Among them, the transcatheter mitral valve-in-valve implantation (TMViV) is the most matured strategy which has obtained US Food and Drug Administration approval and European Union CE Mark and may be considered as an alternative to surgery in patients at high or inoperable surgical risk according to current guidelines [3, 4]. The most frequently used transcatheter heart valves (THVs) are the balloon expandable valves during the TMViV procedures. Cheung *et al*. [5] performed the first successful human implantation of a THV in a degenerated bioprosthetic mitral valve via transapical approach in 2009. And the delivery approach in most patients had been transapical in early stage due to its technical easiness [6]. However, transapical approach has been associated with an increased risk for periprocedural complications and mortality and a slower recovery [1, 2, 7, 8, 9]. Over the past few years, the transseptal approach has been increasingly adopted by more operators. This fully percutaneous approach has shown a safe and effective procedure with rapid recovery. Additionally, preliminary data from the VIVID registry showed that left ventricular function in patients with ejection fraction <50% at baseline had greater improvement in patients treated with transseptal versus transapical TMViV [10, 11]. The transseptal procedure, however, is technically challenging.

The two main technical challenges of transseptal approach are the pursuit of proper transseptal puncture position [12] and ideal coaxiality for THV system delivery [13, 14]. Some patients have an atrial septal incision during the first mitral valve surgery. These patients generally have a scarred or thickened septum due to the prior surgery, which makes it difficult to puncture the atrial septum. In this case, the septum could be punctured with radiofrequency transmitted through the transseptal needle. Then, atrial septostomy with large peripheral balloon expanding are also required to obtain sufficient septal defect [15, 16, 17]. Even so, mounted valve crossing the septum may be a challenging step, and even serious troublesome situations such as valve displacement and support guide wire detachment into the left atrium. Addressing these issues may prolong the procedure and make it more difficult. Meanwhile, the crossing of the mitral orifice may be more challenging because the THV may block against the bioprosthetic ring or calcific bulks of the mitral annulus because of the obliquity of the THV regarding the mitral orifice. Poor coaxiality may also lead to THV migration or paravalvular leaks post deployment [18]. These problems may eventually require more complicated strategy or even open surgery to resolve.

Our team used a long pre-curved sheath in TMViV procedure, which significantly simplified the procedures. This method avoids difficulties when the loaded THV passes through the atrial septum and mitral valve. At the same time, it can effectively avoid large iatrogenic atrial septal defect, and the delivery system can easily reach the optimal anchoring zone and achieve ideal catheter coaxiality. If the delivery system is difficult to reach the desired deployment position, it can also be fully retracted into the long sheath. This method is expected to improve the efficiency of procedure and reduce the difficulty of procedure. The objective of this study was to evaluate the effectiveness of long pre-curved sheath techniques in TMViV procedures.

## 2. Methods

### 2.1 Patient Population 

The study protocol was approved by the institutional ethics review board of Xijing Hospital (Approval Number: QX20191018-2). Between January 2020 and December 2021, 27 patients with degenerated bioprosthetic mitral valve underwent transcatheter mitral valve in valve (TMViV) procedures using a balloon-expandable valve. The selected 27 patients were consecutive patients in the department of Cardiovascular Surgery, Xijing Hospital.

All cases were discussed by the heart team. Patients were selected for the TMViV candidates based on preoperative risk assessment [Society for Thoracic Surgeons (STS) score ≥8.0 or EuroSCORE II ≥8.0], the presence of comorbidities, the previous surgical interventions, frailty, and general clinical conditions. Exclusion criteria for the TMViV procedure were active endocarditis, presence of prosthetic valve thrombosis or thrombus in the left ventricle and paravalvular regurgitation as the mechanism for mitral insufficiency. Thrombus in the left atrial appendage was considered a relative contraindication and evaluated individually. All patients or guardians of the patients provided informed consent to participate in the study, and all clinical documents were reviewed for analysis. The patients were advised of the procedural risks and options as well as of the off-label use of the TMViV devices.

**Fig. 1.**
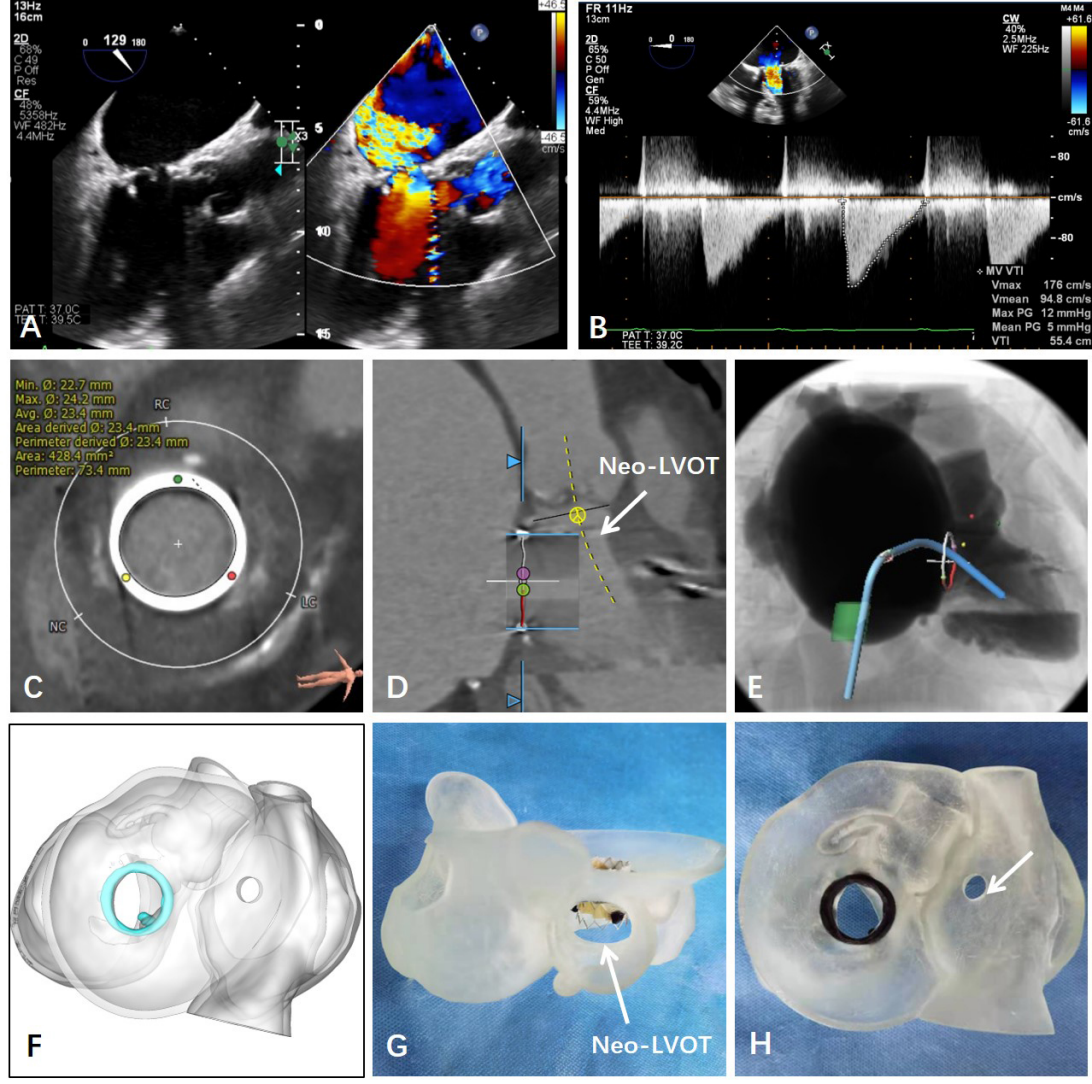
**Preoperative measurements based on TEE, CT and 3D printing**. (A) Preoperative ultrasonic measurement on the degenerated bioprosthetic mitral valve. (B) Mitral regurgitation was measured by 3D TEE. (C) CT data were used to reconstruct and measure the valve ring before the intervention. (D) Left ventricular outflow tract (LVOT) was measured. (E) Preoperative planning of the interventional path. (F–H) Preoperative 3D printing model and individualized simulation of patients’ mitral valve.

Patient demographics and medical histories are shown in Table 1. All patients were diagnosed with transthoracic echocardiography (TTE) before the procedures. For patients with complex anatomical structure, transesophageal echocardiography (TEE) was conducted. The dimension of the prosthetic mitral valve annulus, left ventricle, left atrium, and left ventricular outflow tract (LVOT) were measured based on the preoperative computed tomography angiography (CTA). The measurement data guide procedural strategy and valve size selection. All patients’ individual three dimensional (3D) printing models of the left heart were made based on X-ray computerized tomography (CT) data in order to help the operator to observe the anatomy accurately in the standard technique, as previously described [19] (Fig. 1).

**Table 1.** **Preoperative clinical characteristics**.

Variables		Group A (n = 10)	Group B (n = 17)	*p *value
Gender, male		3 (30.0%)	5 (29.4%)	0.2315
Age, years		69.2 ± 9.3	72.6 ± 6.1	0.4832
Weight, kg		65.2 ± 6.7	60.8 ± 4.2	0.7241
Time since mitral valve replacement, years		9.8 ± 2.9	10.8 ± 3.7	0.0663
Mechanisms of failure				0.2479
	Stenosis	2 (20.0%)	4 (23.5%)	
	Regurgitation	5 (50.0%)	9 (53.0%)	
	Combined stenosis and regurgitation	3 (30.0%)	4 (23.5%)	
Previous mitral valve type				0.0810
	Medtronic Hancock II	5 (50.0%)	9 (52.9%)	
	Edwards Perimount	1 (10.0%)	3 (17.6%)	
	Medtronic Mosaic	0	3 (17.6%)	
	Carpentier-Edwards	2 (20.0%)	2 (14.8%)	
	St Jude Epic	1 (10.0%)	1 (5.9%)	
Comorbidities				0.5343
	Atrial fibrillation	7 (70.0%)	12 (70.6%)	
	Coronary artery disease	2 (20.0%)	5 (29.4%)	
	Diabetes	1 (10.0%)	3 (17.6%)	
	Stroke	1 (10.0%)	2 (11.8%)	
	Systemic hypertension	2 (20.0%)	3 (17.6%)	
	Pulmonary hypertension	8 (80.0%)	14 (82.4%)	
	COPD	1 (10.0%)	2 (11.8%)	
	Chronic renal insufficiency, Creatinine >1.5 mg/dL	1 (10.0%)	1 (5.9%)	
Previous combined procedure				0.7295
	Aortic valve replacement	0	1 (3.7%)	
	CABG	2 (20.0%)	3 (17.6%)	
	Tricuspid valve repair	7 (70.0%)	12 (70.6%)	
	LVEF			0.0698
	<40	3 (30.0%)	3 (17.6%)	
	40–50	4 (40.0%)	8 (47.1%)	
	>50	3 (30.0%)	6 (35.3%)	
NYHA FC				0.1498
	NYHA FC I	0	0	
	NYHA FC II	1 (10.0%)	1 (5.9%)	
	NYHA FC III	3 (30.0%)	6 (35.3%)	
	NYHA FC IV	6 (60.0%)	10 (58.8%)	
STS Score				0.9216
	0–4	0	0	
	5–8	2 (20.0%)	3 (17.6%)	
	>8	8 (80.0%)	14 (82.4%)	

Categorical variables are presented as frequency (%); continuous variables are presented as mean ± standard deviation when normally distributed. COPD, chronic obstructive pulmonary disease; CABG, Coronary artery bypass grafting; LVEF, left ventricular ejection fraction; NYHA FC, New York Heart Association functional class; STS, society for Thoracic Surgeons.

### 2.2 Procedural Details

All TMViV procedures were performed in the hybrid catheterization laboratory. All procedures were performed via the transfemoral transseptal approach under general anesthesia. Pre-procedural work-up was completed according to the institutional guidelines. CTA data was used for the accurate assessment of native bioprosthetic mitral valve anatomy, left ventricle, left atrium, and LVOT, and to aid prosthetic valve sizing and septal puncture planning. Valve sizing for native bioprosthetic mitral valve was based on the area- or perimeter-derived mean diameter on CTA measurements by using the largest annular diameter in systole. The size of TMViV valve were selected based on the measured diameter approximately 8% to 15% oversizing. For individual patient, the valve-in-valve app was additionally used for sizing of the transcatheter prosthesis prior to procedure (http://www.ubqo.com/vivmitral).

All patients were treated by implantation of the Prizvalve™ prosthesis (Newmed, Shanghai, China). The Prizvalve™ transcatheter valve is made of a balloon-expandable nickel-chromium frame and tri-leaflet bovine pericardial valve. As a part of the prosthesis, the inner and outer polyethylene glycol terephthalate (PET) skirt at the inflow tract is designed to reduce postprocedural perivalvular leakage (PVL). The leaflets have anti-calcification treatment. Low density and large cells at the outflow part are designed to provide sufficient blood flow. The valve prosthesis is manufactured in four different sizes (20, 23, 26, and 29 mm). A 14/16F expandable sheath is utilized for low-profile delivery. In this study, we also tried a long pre-curved sheath which has a hemostatic valve (Hunan ATP Medical Instrument Co., Ltd, Xiangxiang, Hunan, China) combined with Prizvalve™ delivery system. The long pre-curved sheath is 75 cm long with 22F profile. The tip of the sheath is pre curved with a 2 cm long and 45° bend. During the TMViV procedure, the long pre-curved sheath can directly cross the atrial septum and reach the position of the mitral annulus, which avoiding excessive expansion of septum and establish a safe advancing approach for the delivery system. Thereafter, the balloon-expanding valve system can be advanced smoothly cross the atrial septum and bioprosthetic ring or calcific bulks of the mitral annulus. The long pre-curved sheath can also make delivery system achieve better release positioning and alignment with the bioprosthetic ring during deployment. In this study, the regular 14/16F expandable sheath were used for low-profile delivery in the early 10 cases, and 22F long pre-curved sheath were used in the subsequent 17 cases.

During all procedures, TMViV was guided by real-time TEE and fluoroscopy. Unfractionated heparin was administered to maintain an activated clotting time above 250 seconds. A temporary pacemaker was placed in the right ventricular apex via femoral vein at the beginning of the procedure, which can provide rapid pacing with the induction of slow flow through the mitral valve during transcatheter valve implantation. All procedures were performed using an antegrade transseptal approach via right femoral vein.

Transseptal puncture was performed under fluoroscopy and TEE guidance in a middle and posterior localization of the septum. We usually chose the central point of the bioprosthetic ring on the right anterior projection as the reference for the height of the puncture. Meanwhile, the posterior puncture would be preferred under the guidance of TEE and the puncture site was located in the posterior part of the oval fossa. Generally, this puncture spot is about 3 cm away from the plane of the mitral valve anulus.

After transseptal puncture, the mitral bioprosthesis was crossed with hydrophilic guidewire or a standard 0.035-inch J-guidewire over a steerable guiding catheter (Agilis, St. Jude Medical, USA). The degree of the curve was 45° and the size of the Agilis catheter was 8.5 Fr. Afterwards, the standard wire was exchanged for an extra stiff wire with its end manually bended as a pigtail-curve placed in the left ventricular apex (e.g., Amplatz Super Stiff Wire) over a standard 5F pigtail catheter. Then, a balloon dilatation of the interatrial septum was performed using an Atlas Gold 12 to 14 mm × 40 mm Balloon catheter (Atlas, BARD Medical, USA). The 10 mm × 40 mm Balloon catheter were used if the long pre-curved sheath advanced thereafter. No balloon valvuloplasty of bioprosthesis was performed in this series of cases prior to TMViV.

In first 10 cases, 14/16F expandable sheath were advanced via the stiff guidewire then. Afterwards, the delivery system with the mounted Prizvalve™ prosthesis was carefully inserted via stiff guidewire into the left atrium and into the mitral valve under maximal flexion of the delivery system. The valve system was double-checked about valve mounted in the opposite direction as performed for transfemoral transcatheter aortic valve replacement (TAVR) before inserting into the sheath. If some resistance occurs during delivery system crossing the septum or bioprosthetic ring, the catheter should not be pushed forcefully, but removed into the right atrium or left atrium and another attempt should be made using a different orientation of the catheter. These manipulations require experience from the operator.

If the Prizvalve™ prosthesis was delivered into mitral bioprosthesis, careful adjustment should be made to make the valve aligned inside the bioprosthetic ring and the lower marker of the valve located at the anulus plane with both TEE and fluoroscopic guidance. Then, the Prizvalve™ prosthesis was deployed under rapid ventricular pacing (160–180 beats/min). Satisfactory positioning and function were confirmed by TEE and fluoroscopy. Satisfactory position of the prosthesis means its outer skirt exactly placed into the valvular plane of the bioprosthesis ring, which was achieved by a slight protrusion of approximately 10–20% of the prosthesis into the left atrium. Postdilation would only be considered if the new prosthesis was under-expanded or para-valvular leak was present.

**Fig. 2.**
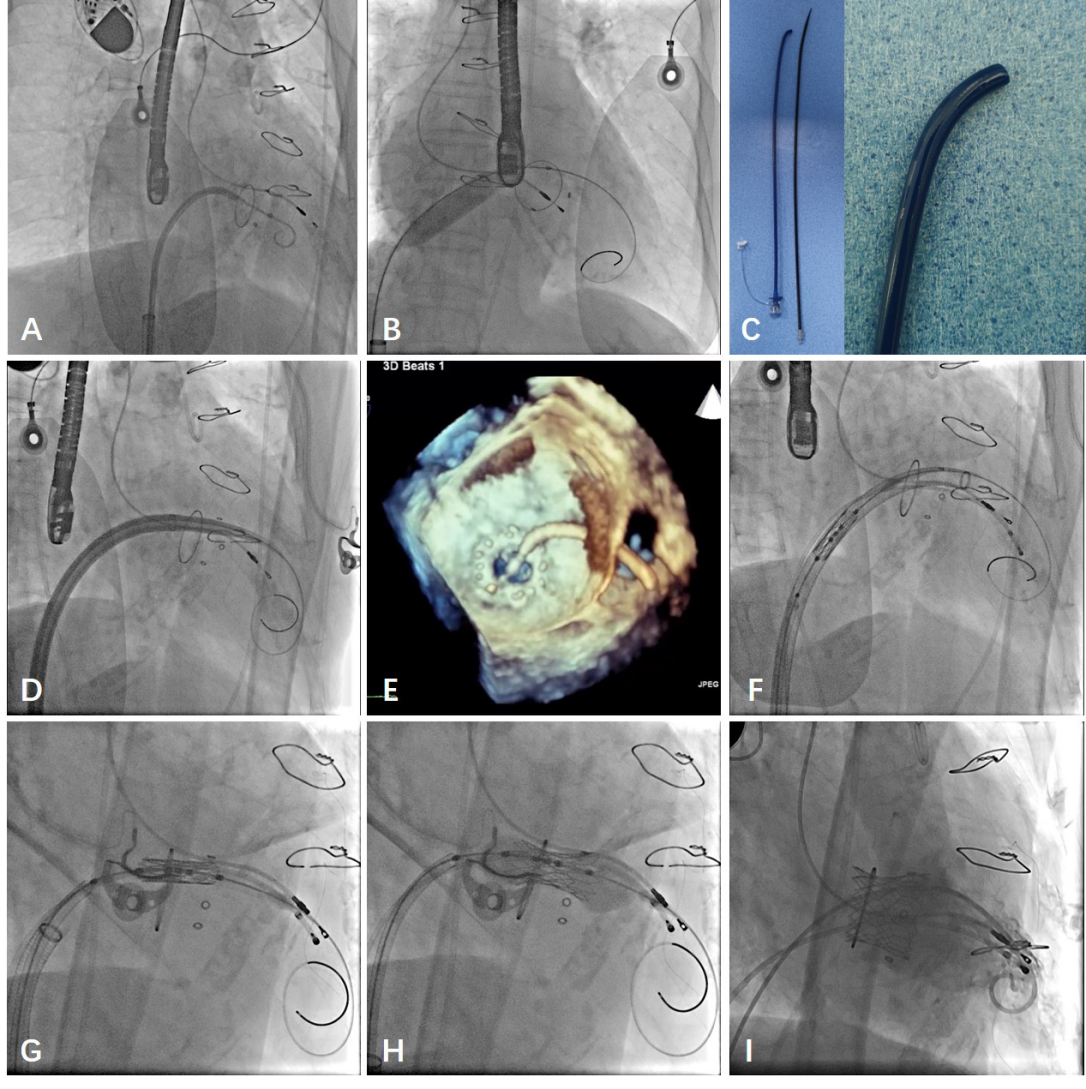
**Procedural details with long pre-curved sheath**. (A) The catheter was passed through the puncture site of atrial septum and crossed the mitral valve. (B) The Lunderquist guide wire was replaced to establish the track and balloon dilatation was performed. (C) The long pre-curved sheath. (D) The long pre curved sheath was placed at the mitral valve plane along Lunderquist guide wire. (E) It was measured by 3D TEE to reconfirm that the long pre curved sheet passed through the mitral valve plane. (F) The Prizvalve ™ system was delivered along Lunderquist guide wire. (G) The valve was place at the predetermined release position of the mitral valve plane. (H) The Balloon dilatation was performed to release the valve. (I) After the valve was completely released, the function and position of the valve were measured by DSA angiography.

In rest 17 cases, the 22F long pre-curved sheath were used to deliver the valve system. Prior to transseptal insertion of the Prizvalve™ prosthesis delivery system into the left atrium, the 22F long pre-curved sheath were advanced via the stiff guidewire cross the septum and bioprosthetic ring into the left ventricle directly. It was much easier with stiff dilator than unsheathed delivery system. Then the Prizvalve™ prosthesis was delivered into mitral bioprosthesis via the long pre-curved sheath smoothly without any kinking on the septum or bioprosthetic ring. Thereafter, the long pre-cured sheath was retrieved to a safe position, if the prosthesis was aligned inside the bioprosthetic ring. Other subsequent strategies are the same as first 10 cases (Fig. 2).

The procedural success was defined as the ability of the device to be deployed as intended and the delivery system successfully retrieved without procedural mortality or the need for emergency surgery. In general, patients were extubated at the end of the procedure, recovered, and then transferred to the Intensive Care Unit for cardiac monitoring. Routine TTE was performed on the first day after procedure and was repeated before discharge. Then, patients were discharged on dual antiplatelet therapy, or on anticoagulation with warfarin.

### 2.3 Data Collection

Preoperative and postoperative data were collected prospectively. All clinical files were reviewed, and perioperative characteristics were documented, including procedural time, fluoroscopic time and postoperative hospital stay, etc. All patients were seen in the clinic to ascertain their clinical status (New York Heart Association functional class) and adverse events after discharge. Transthoracic echocardiography was performed to evaluate the improvements in the construction and function of the patients’ hearts at 30 days, 6 months, 1 year and yearly thereafter. Computed tomography angiography was also performed during the follow-up period in some patients. To further scrutinize our data, we divided the group into the first 10 patients using 14/16F expandable sheath (Group A) and the rest 17 patients with long pre-curved sheath (Group B) in order to assess the impact of the different sheath and procedural details on outcomes.

### 2.4 Statistical Analysis 

Statistical analysis was conducted with SPSS 22.0 software (IBM SPSS Statistics for Macintosh, Version 22.0. IBM Corp, Armonk, NY, USA). Continuous variables are presented as means ± SD, and categorical variables are expressed as percentages. Univariable comparisons have been performed with the Shapiro-Wilk test. Variables that are not normally distributed should be presented as median with interquartile range and be compared using the Mann Whitney U test. Values of *p <* 0.05 were considered statistically significant.

## 3. Results

### 3.1 Procedural and In-Hospital Outcomes

The procedural success rate was 96.3% in all 27 patients. Procedural characteristics are summarized in Table 2. All procedures were performed with right femoral vein access, transseptal puncture and placement of a balloon expandable Prizvalve™ prosthesis as described above. Seventeen patients received 26 mm prostheses; the remaining ten patients received 29 mm prostheses. Post balloon dilatation was performed in one case. There were no hospital deaths. The iatrogenic atrial septal defect (ASD) closure was performed in 2 patients (7.4%) for ASD over 10 mm or bidirectional interatrial shunt. Total procedure time was (96.1 ± 28.2) min, the fluoroscopic time was (27.4 ± 6.5) min, and total contrast volume was (50.7 ± 10.1) mL.

**Table 2.** **Procedural and postprocedural characteristics**.

Variables	Group A (n = 10)	Group B (n = 17)	*p* value
Procedural success	10 (100%)	17 (100%)	/
Device size			0.7196
26 mm	6 (60.0%)	11 (64.7%)	
29 mm	4 (40.0%)	6 (35.3)	
Pre-dilatation	0	0	/
Post-dilatation	1 (10.0%)	0	/
Procedural time, min	115.2 ± 25.6	85.2 ± 24.3	0.0048
Fluoroscopic time, min	31.3 ± 5.1	24.3 ± 5.2	0.0073
Contrast dose, mL	51.0 ± 9.7	48.8 ± 9.8	0.5815
ASD closure	2 (20%)	0 (0%)	0.1282
Procedural complications			
Hemorrhage need blood transfusion	1 (10.0%)	0	/
Permanent pacemaker implantation	1 (10.0%)	0	/
Transfer to arteriovenous loop approach	1 (10.0%)	0	/
In-hospital mortality	0	0	/
Extubate in the catheterization laboratory	4 (40.0%)	1 (5.9%)	0.0473
ICU-stay, hours	20.0 ± 2.9	19.1 ± 2.4	0.3981
Post-procedural hospital-stay, days	5.7 ± 2.3	5.5 ± 2.0	0.8416
NYHA FC at POD 30			0.0962
NYHA FC I	2 (20.0%)	4 (23.5%)	
NYHA FC II	6 (60.0%)	10 (58.8%)	
NYHA FC III	1 (10.0%)	3 (17.6%)	
NYHA FC IV	1 (10.0%)	0	
Readmission within 30 days	2 (20.0%)	0	/

Categorical variables are presented as frequency (%); continuous variables are presented as mean ± standard deviation when normally distributed. ASD, atrial septal defect; ICU, Intensive Care Unit; NYHA FC, New York Heart Association functional class.

Twenty-two patients were extubated in the hybrid catheterization laboratory and the other five patients were transferred to the Intensive Care Unit for recovery post procedure. One patient received blood transfusion because of hemorrhage at the femoral puncture in the Intensive Care Unit. One patient had received a permanent pacemaker due to high-degree atrioventricular block at postoperative day 3. In one case, the delivery system kinking at the septum and could not enter the left atrium after careful manipulations of the catheter. This case was performed with a 14/16F sheath. Then the transapical puncture was performed to establish an arteriovenous loop with a snare technique. The delivery system was advanced into the bioprosthetic ring via the arteriovenous loop guidewire and the new prothesis was deployed satisfactorily. All these patients recovered before discharge from the hospital. There were no other major post-procedural complications and the median length of hospital stay was 5 days.

**Fig. 3.**
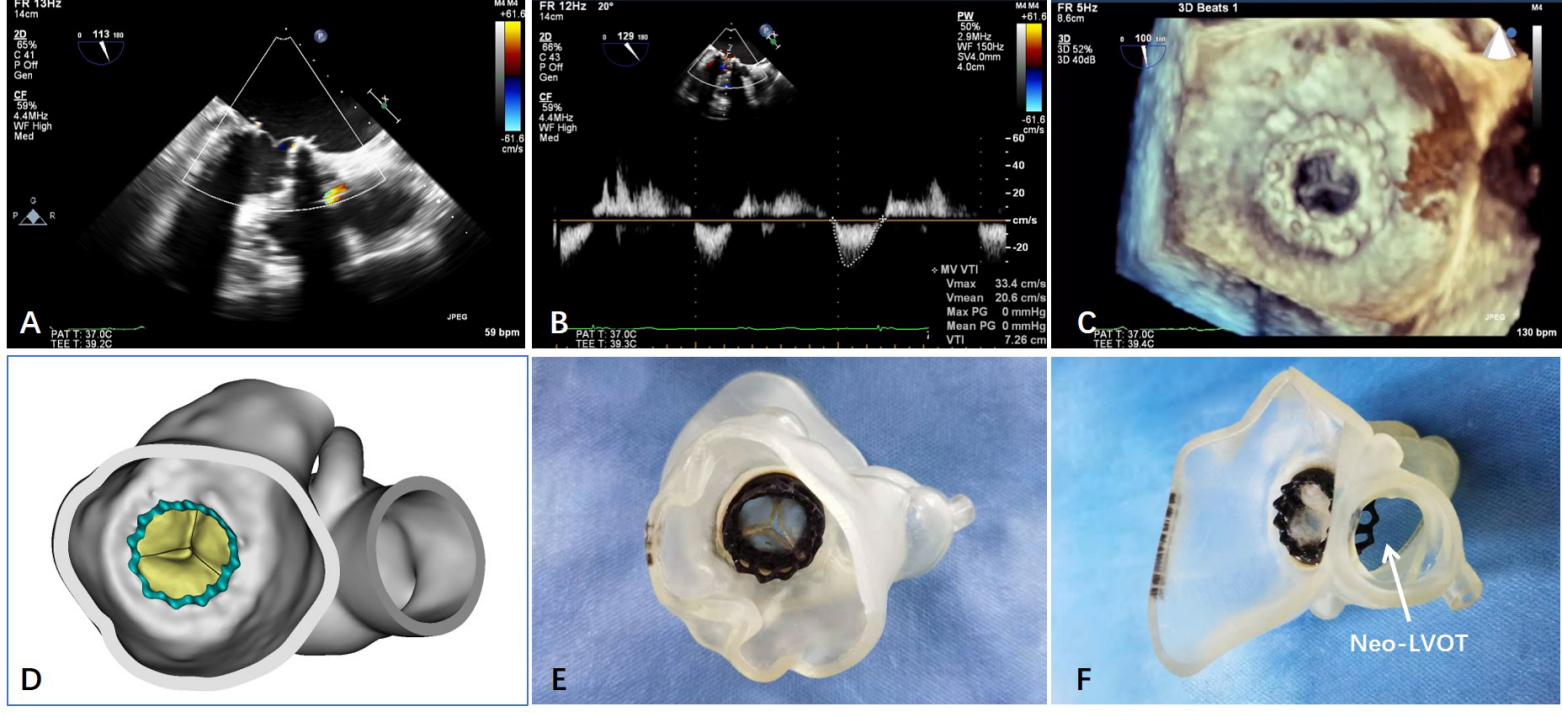
**Postoperative transthoracic echocardiograms, CT scans and 3D printing model**. (A) Less than trivial regulation was found by 3D TEE. (B) There was no LVOT observation diagnosed with a newly observed flow maximum of >150 cm/s in pulsed wave Doppler measurement after TMViV. (C) The position and function of the valve were reconstructed and observed by 3D TEE. (D–F) The anatomical structure of mitral valve was reconstructed by 3D printing technology after TMViV.

Post-procedural transthoracic echocardiograms were performed in all patients the day after the procedure. All showed a well seated valve; the average mean gradient was (3.5 ± 1.0) mmHg. Twenty-five patients had no or less than trivial regurgitation. Two patients had mild paravalvular regurgitation post-procedure relating to the pre-existing leak around the bioprosthetic valve. There was no LVOT obstruction diagnosed with a newly observed flow maximum of >150 cm/s in pulsed-waved Doppler measurement after TMViV (Fig. 3). No events such as stroke and myocardial infarction took place in the cohort.

A subsequent sub analysis of both groups did not show significant differences between groups regarding preoperative risk scores and baseline characteristics. However, there were shorter procedural time [(85.2 ± 24.3) vs. (115.2 ± 25.6) min, *p* = 0.0048] and shorter fluoroscopic time [(24.3 ± 5.2) vs. (31.3 ± 5.1) min, *p* = 0.0073] in pre-curved sheath group than ones in regular sheath group. In one case in regular sheath group, the delivery system could not normally enter the left atrium through the septum. Then, the procedure was completed by the conversion from the transseptal approach to the arteriovenous loop approach. Meanwhile, two patients in regular sheath group (Group A) received transcatheter atrial septal defect closure after new prothesis deployment during the procedures because of the large right to left transeptal shunt. While no patient needed intraoperative atrial septal defect closure because of excessive residual atrial septal defect in pre-curved sheath group (Table 3).

**Table 3.** **Comparation of the procedural characteristics between regular expandable sheath (Group A) and the pre-curved long sheath (Group B)**.

	Group A (n = 10)	Group B (n = 17)	*p* value
Procedural time, min	115.2 ± 25.6	85.2 ± 24.3	0.0048
Fluoroscopic time, min	31.3 ± 5.1	24.3 ± 5.2	0.0073
Contrast dose, mL	51.0 ± 9.7	48.8 ± 9.8	0.5815
ASD closure	2 (20%)	0 (0%)	0.1282
Extubate in the catheterization lab laboratory	4 (40%)	1 (5.9%)	0.0473
ICU-stay, hours	20.0 ± 2.9	19.1 ± 2.4	0.3981
Post-procedural hospital-stay, days	5.7 ± 2.3	5.5 ± 2.0	0.8416

Categorical variables are presented as frequency (%); continuous variables are presented as mean ± standard deviation when normally distributed. ASD, atrial septal defect; ICU, Intensive Care Unit.

### 3.2 Follow-Up

**Fig. 4.**
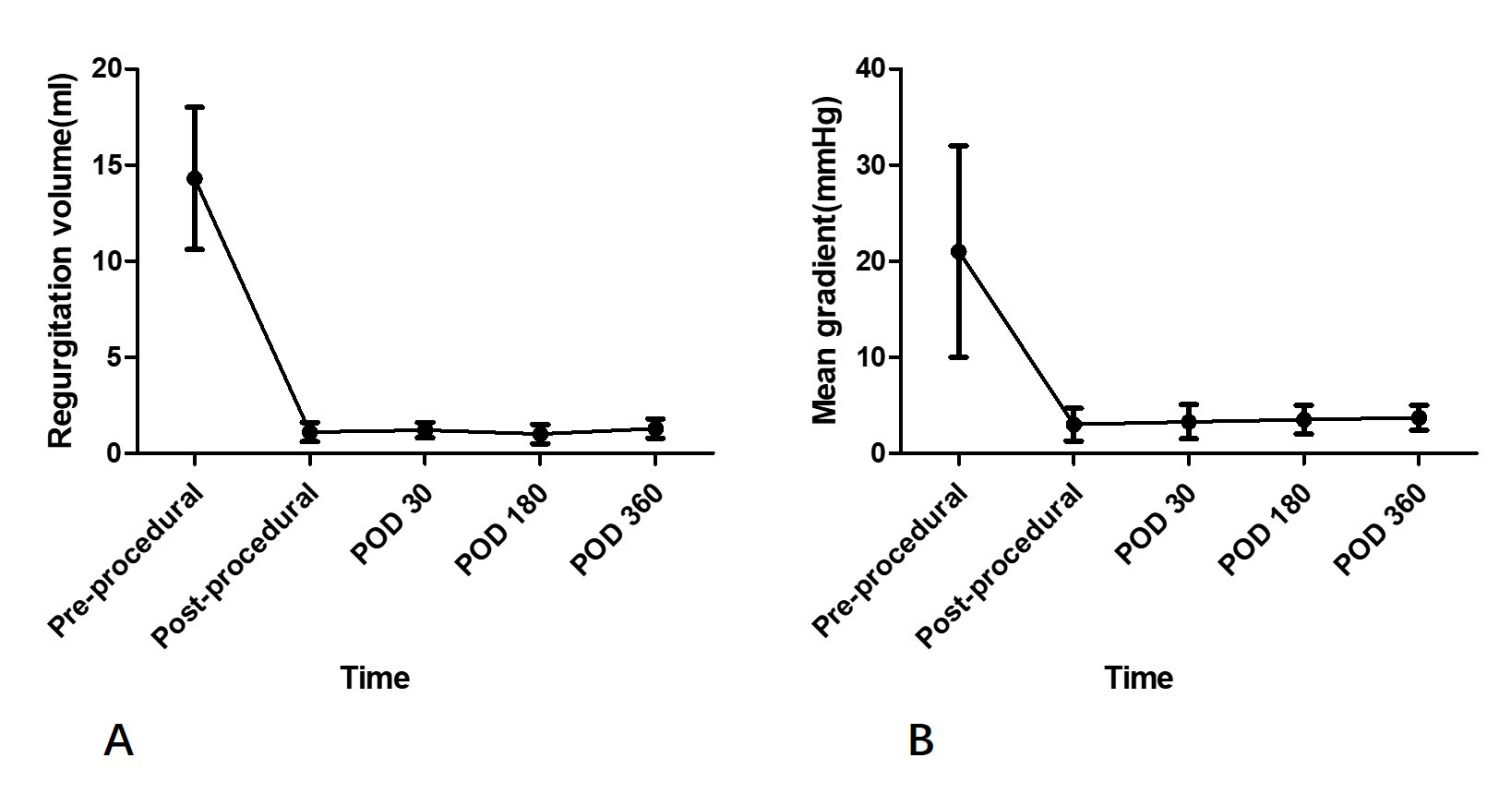
**Transvalvular regurgitation and gradients on echocardiography during 1-year follow-up**. (A) Regurgitation volume for patients with previous regurgitation because of degenerated bioprosthesis. (B) Mean gradient for patients with previous stenosis because of degenerated bioprosthesis. POD, post operation days.

The median follow-up period was 12 (1–21) months, and follow-up was 100% completed. After hospital discharge, no death occurred during 30 days follow-up in both groups. Nine (90.0%) patients in Group A and sixteen (94.1%) patients in Group B improved by ≥1 New York Heart Association (NYHA) functional class at 30 days. Meanwhile, eight (80.0%) patients in Group A and fourteen (82.4%) patients in Group B were in NYHA functional class ≤II. Most patients’ heart failure symptoms improved considerably. Two patients in regular sheath group (Group A) were readmitted to the hospital with non-specific chest discomfort within 30 days post discharge; all investigations were negative and they were discharged home without further issues. One patient in regular sheath group (Group A) died from the rupture of renal cyst at six months after discharge. One patient in regular sheath group (Group A) was readmitted to a peripheral hospital with severe COPD four times within one year follow-up. No late deaths occurred in this cohort. Clinical improvement according to NYHA functional class remained stable during one-year follow-up in both the two groups. The mean transvalvular gradients fell significantly in patients with stenosed bioprosthetic mitral valve after procedures and was remained throughout the first-year follow-up in both the two groups. And no more recurrent regurgitation was observed during one-year follow-up (Fig. 4).

## 4. Discussions

Bioprosthetic valves are increasingly preferred over mechanical valves, due to avoiding lifelong anticoagulation and the lower risk of thromboembolic complications [20]. At the same time, the aging of the population has led to an increase in elderly patients with valvular disease, and the amount of bioprosthetic valves will further increase. However, bioprosthetic valves have a limited durability and valve deterioration is frequently observed [21, 22]. It is estimated that the number of patients requiring re-treatment for bioprosthesis failure is likely to rise within the next years. Redo open heart surgery for bioprosthetic valve failure is associated with significant risks, particularly in patients with comorbidities, including advanced age [23]. It is not only a challenge of surgical techniques and prognosis, but also a huge challenge of patient psychological acceptance. TMVI has been investigated in 4 different settings: valve in native, valve-in-valve (VIV), ViR and ViMAC. VIV is the most promising setting for TMVI [24, 25]. Over the past decade less invasive transcatheter valve-in-valve (VIV) procedures have been increasingly utilized in the aortic, mitral, pulmonary, and tricuspid positions [26, 27, 28, 29, 30, 31]. It has been considered as an alternative to surgery in patients at high or prohibitive surgical risk according to latest guidelines. Transcatheter valve-in-valve procedure is a revolutionary technological innovation. This treatment has the obvious advantages of not requiring open chest, cardiopulmonary bypass, cardiac arrest, and short procedural time, minimal trauma, and fast recovery [29, 32, 33]. In this cohort of patients, all 27 cases achieved procedural success. The procedural duration is only about 90 minutes, and the postoperative hospital stay is only less than 5 days. There are even patients who complete all treatments within 24 hours and are safely discharged after receiving day surgery. At the same time, the follow-up results are encouraging. Except for one patient who died of non-cardiac-related causes six months after procedure, all other patients survived at 1 year follow-up. And there were no re-hospitalizations for serious complications in the whole group. The postoperative recovery of cardiac function in this cohort is also encouraging. 92.6% of patients had a postoperative improvement by ≥1 New York Heart Association (NYHA) functional class. This result is similar to those of the other previous studies [30, 31, 33, 34, 35], reflecting the significant advantages of TMViV procedure as a minimally invasive treatment technique in the perioperative period. At the same time, the results of short-term follow-up are not inferior to those of traditional surgery. Moreover, the medium-term clinical outcome of this technology is also not inferior to redo surgery according to the 3-year follow-up data from the VIVID Registry [30]. Therefore, both the 2020 ACC/AHA Valvular Disease Management Guidelines and the 2021 ESC/EACTS Guidelines suggested the treatment of transcatheter valve-in-valve (VIV) procedures as class IIa recommendation for patients at high or prohibitive surgical risk [3, 4].

TMViV procedure was first implemented by transapical approach [5], and most patients underwent transapical procedures in the early stages [6]. This approach facilitates coaxial delivery of the THV across the failed bioprosthesis and is technically less challenging than the transseptal alternative [36, 37, 38, 39]. And many centers are familiar with this technique due to their experience in transapical TAVR. There were also a few case reports in which THVs were implanted via trans-atrial approach with left minimal thoracotomy. Although the transapical approach is most commonly used previously due to its technical easiness, it has been associated with an increased risk for periprocedural complications and mortality and a slower recovery. Therefore, TMViV via the transseptal approach might be a better option for this high-risk population. At present, percutaneous transseptal TMViV has become the preferred least invasive treatment choice for patients with degenerated mitral bioprosthesis [40, 41]. However, it must be noted that the transseptal approach is more technically challenging than transapical approach. Therefore, guidelines also explicitly recommend that such treatment be done in experienced institutions [3].

Transseptal TMViV procedure has two technical challenges. One is transseptal puncture technique at scarred and calcified atrial septum and choosing the appropriate puncture position. The other is to establish an ideal delivery track that facilitates the THV system to across the atrial septum and failed bioprothesis, and reach the perfect release position. The key point of transseptal puncture technique is to choose the appropriate puncture position, which facilitates the ideal delivery track. Under TEE and fluoroscopic guidance, transeptal puncture is usually performed at the predetermined location from CT measurements. TMViV procedures generally prefer transseptal puncture in the inferoposterior portion of atrial septum instead of superoposterior location of septostomy similar to the transseptal puncture for MitraClip procedures. The puncture is usually located in the middle of the fossa ovalis and approximately 3 cm high from the mitral valve plane at a TEE four-chamber view. This location is similar to transseptal access for percutaneous mitral balloon commissurotomy. Our institution usually uses the center of the mitral prosthesis as a marker of puncture height on fluoroscopy in the projection perpendicular to the plane of the mitral bioprosthesis. It is more difficult to navigate the septum and deliver the THV with superior punctures. And the inferior puncture is not conducive to the ideal coaxiality of the catheter in the mitral prothesis [9, 12, 13]. The other technical challenge of transeptal puncture is resistant septum. It might be difficult for some patients to succeed in routine puncture. These patients generally have a scarred or thickened septum from the prior surgery. If resistance to crossing the septum with the needle occurs, pressure must be continuously applied until crossing. When crossing is still not possible, radiofrequency may be used by a standard electrosurgical cautery generator via the puncture needle, brief pulses being applied to the hub of the needle by direct contact [12, 13]. If these methods are still ineffective, you may need to re-select the puncture position. Once the transseptal puncture succeed, it is necessary to apply a sufficiently large balloon for atrial septostomy multiple times in order to obtain a sufficiently loose transseptal channel. Therefore, many patients will have obvious residual iatrogenic atrial septal defects after procedures.

Even if the balloon dilatation of atrial septum is successfully completed, it may still be challenging for the THVs delivery system to pass through the septum. Such a situation is not uncommon in transseptal TMViV procedures. The edge of scarred and calcified septum may lead to entrapment and blockage of the THV. If some resistance occurs, pushing catheter forcefully is a very dangerous, which may lead to valve dismounting/deformation, or ventricular rupture caused by excessive force of the stiff support guide wire, or guide wire inadvertent pullback into the left atrium or even loses of the established transseptal access. Addressing these situations requires a wealth of experience from the operator. The operator should carefully manipulate the catheter by torquing the system and adjusting the flexion angle until crossing the scarred atrial septum. If crossing is still not possible or guide wire pullback into the left atrium, the THV should be reintegrated into the sheath and removed and started all over again. At this point, complete withdrawal of the THV may also encounter greater difficulties, as balloon expanding valves are often difficult to retract into the regular delivery sheath. In addition, even if the THV successfully crosses the atrial septum, the crossing of the mitral orifice may be more challenging because the THV may block against the bioprosthetic ring or calcific bulks of the mitral annulus, or in severely stenotic orifices. In most cases, the undesirable catheter coaxiality and the obliquity of the THV with regard to the mitral orifice is the main cause of blockage. These will cause great difficulties in the continuation of the procedure.

The long pre-curved sheath we used in this study can effectively solve the above technical difficulties. After successfully transeptal puncturing and establishing a track to left ventricular apex, the long pre-curved sheath was advanced directly into the left ventricle along the stiff guide wire. Once the long sheath crossed the septum and bioprosthetic ring, the ideal THV delivery track was already established. Thereafter, the mounted THV system was advanced smoothly through the atrial septum and bioprosthetic ring within the long sheath, so that no blockage occurred in the atrial septum and bioprosthetic ring. At the same time, due to the strong support of the long pre-curved sheath, the coaxiality of the delivery track will be more stable. And it is easier to manipulate the optimal orientation of the catheter by rotating sheath. Before THV deployment, the retracted long sheath can still support the delivery system to position the THV appropriately. The curvature at the tip of the long sheath helped to maintain the sagittal position coaxiality of the THV within the bioprosthetic ring. Even in cases where a complete withdrawal of THV delivery system is required, it can be easily retracted into the 22F sheath, avoiding the irreversible condition of unsheathed procedure. In addition, there is no need to dilate atrial septal with an oversized balloon after puncture. It is relatively easy for the long sheath system with dilator to cross the calcified atrial septum under the support of stiff guide wire. For patients who had not undergone atrial septal incision and suturing in the previous surgery, even balloon dilatation is not required, which is similar to the Mitraclip procedure. Therefore, there is generally no excessive iatrogenic atrial septal defect after procedure, and there is no need for closure. In this study, the long pre-curved sheaths were used in the TMViV procedures for the later 17 patients. Compared with previous procedures done with regular sheaths, this technique significantly saved procedural time and fluoroscopic time, and improved surgical efficiency. And none of the patients required postoperative closure of the atrial septal defect. However, in the first 10 patients with regular expandable sheaths, two had transcatheter atrial septal defect closure due to large iatrogenic interatrial shunt. This increases the procedural duration and medical costs with more implanted intracardiac devices.

One of the most important features limiting the use of TMVI is LVOT obstruction. The structure of Prizvalve™ valve is similar to that of other commercial spherical expansion valves, and the screening criteria are also similar. In this study, we have followed the consensus standards of the past. The number of cases enrolled in our study was small, and most of them were mainly reflux cases. The patients had large left ventricle. The assessment before intervention showed that there was no risk of left ventricular outflow tract obstruction. All patients successfully received valve in valve implantation, and no such complication occurred after TMVI.

## 5. Limitations

The present series is a retrospective, non-randomized study in a single center with its inherent limitations. The relatively small number of patients did not allow us to find more convincing conclusions.

## 6. Conclusions

TMViV procedure is currently an alternative treatment for patients with degenerated mitral bioprothesis at high or inoperable surgical risk. The advantages of this minimally invasive treatment reflect the revolutionary technological innovation of transcatheter valvular therapy. Transseptal TMViV procedure with a long pre-curved sheath avoids the blockage of the THV delivery system in the scarred and calcified atrial septum and bioprothesis ring, and facilitates the ideal coaxiality required for deployment. The application of long pre-curved sheath in TMViV procedure simplifies transseptal approach and saves procedural time.

## Data Availability

Data and materials are available on request.
